# Structural Influences on Methamphetamine Use Among Black Sexual Minority Men (HISTORY Study): Protocol for a Longitudinal Cohort Study

**DOI:** 10.2196/63761

**Published:** 2024-10-31

**Authors:** Samuel C O Opara, Sabriya L Linton, Brian W Weir, Natalie D Crawford, David P Holland, Antonio Newman Jr, McKinsey Bullock, Marcus O Reed, Srija Dutta, Kamini Doraivelu, Charles Stephens, Justin C Smith, Yeeli Mui, Sophia A Hussen

**Affiliations:** 1 Division of Infectious Diseases Department of Medicine Emory University School of Medicine Atlanta, GA United States; 2 Department of Mental Health Johns Hopkins Bloomberg School of Public Health Baltimore, MD United States; 3 Department of Health, Behavior and Society Johns Hopkins Bloomberg School of Public Health Baltimore, MD United States; 4 Department of Behavioral, Social, and Health Education Sciences Rollins School of Public Health Emory University Atlanta, GA United States; 5 Mercy Care Health Systems Atlanta, GA United States; 6 Hubert Department of Global Health Rollins School of Public Health Emory University Atlanta, GA United States; 7 The Counter Narrative Project Atlanta, GA United States; 8 Positive Impact Health Centers Atlanta, GA United States; 9 Harvard T.H. Chan School of Public Health Boston, MA United States; 10 Department of International Health Johns Hopkins Bloomberg School of Public Health Baltimore, MD United States

**Keywords:** substance use, methamphetamine, social determinants, racism, discrimination, homonegativity, LGBT, mixed methods

## Abstract

**Background:**

Sexual minority men are disproportionately affected by methamphetamine use, with recent studies suggesting an increase in use specifically among Black sexual minority men. Black sexual minority men face unique structural barriers to achieving optimal health. Given its harmful effects, and in light of existing health disparities, an increase in methamphetamine use among Black sexual minority men poses a significant public health concern.

**Objective:**

The Health Impacts and Struggles to Overcome the Racial Discrimination of Yesterday (HISTORY) study is investigating the potential impacts of exposure to the census tract–level structural racism and discrimination (SRD) on methamphetamine use among Black sexual minority men in Atlanta, Georgia, and will identify intervention targets to improve prevention and treatment of methamphetamine use in this population.

**Methods:**

This study uses a mixed methods and multilevel design over a 5-year period and incorporates participatory approaches. Individual-level quantitative data will be collected from a community-based cohort of Black sexual minority men (N=300) via periodic assessment surveys, ecological momentary assessments, and medical record abstractions. Census tract–level measures of SRD will be constructed using publicly available administrative data. Qualitative data collection will include longitudinal, repeated in-depth interviews with a subset (n=40) of study participants. Finally, using a participatory group model–building process, we will build on our qualitative and quantitative data to generate causal maps of SRD and methamphetamine use among Black sexual minority men, which in turn will be translated into actionable recommendations for structural intervention.

**Results:**

Enrollment in the HISTORY study commenced in March 2023 and is anticipated to be completed by November 2024.

**Conclusions:**

The HISTORY study will serve as a crucial background upon which future structural interventions can be built, to mitigate the effects of methamphetamine use and SRD among Black sexual minority men.

**International Registered Report Identifier (IRRID):**

DERR1-10.2196/63761

## Introduction

### Background

Methamphetamine use is increasingly common in the United States. Among the total US population of individuals aged 12 years and older, 1% (or 2.7 million people) reported past-year methamphetamine use in 2022, 66.7% (or 1.8 million) of whom met the criteria for methamphetamine use disorder [1]. These represent the highest rates in an upward trend in past-year methamphetamine use and methamphetamine use disorder, according to the annual National Survey on Drug Use and Health [2]. Sexual minority men are disproportionately affected by methamphetamine use; most recent national surveillance data report rates of methamphetamine use up to 4 times higher among sexual minority men compared to their heterosexual counterparts [3]. Black sexual minority men had historically lower rates of methamphetamine use compared to their White counterparts [4-9]; however, emerging data suggest a changing trend. A 2021 study demonstrated an overall increase in past-year methamphetamine use among Black sexual minority men in New York City from 6.5% in 2004 to 11.3% in 2017, with a concomitant overall decrease in use among White sexual minority men from 17% to 6.2% over the same time frame [5]. A similar trend was demonstrated in Washington, District of Columbia, in 2017 [6], with additional supporting data from qualitative studies and lay publications [10-13].

Methamphetamine is a highly addictive synthetic stimulant drug [14,15] associated with adverse health consequences including, but not limited to, cardiovascular system dysfunction, neurotoxicity, dental decay, mental health comorbidities, polydrug use, fatal overdose, increased sexual risk, HIV and sexually transmitted infection transmission, and decreased health care (including HIV care) engagement [8,15-26]. Methamphetamine use is also associated with social harms including homelessness, food insecurity, exposure to violence, and criminal justice involvement [27-32]. Given the detrimental impacts of methamphetamine use alone, as well as its potential to exacerbate existing health inequities, increasing methamphetamine use among Black sexual minority men constitutes a major public health concern for which effective interventions are urgently needed but sorely lacking.

Research has primarily focused on individual-level determinants of methamphetamine use including social networking, incarceration history, and various forms of victimization (like intimate partner violence and childhood sexual abuse) [4,33-39]. Similarly, interventions have generally focused on individual behavioral change [40-44]. A few interventions have included cultural considerations for Black sexual minority men, focusing on intrapsychic processes and individual-level behavior change to impact methamphetamine use and sexual health [44,45]. While these are critically important, they have not fully addressed structural barriers that contribute to methamphetamine use or explore the intersecting social positions occupied by Black sexual minority men and the resultant synergistic manifestations of structural racism and discrimination (SRD) to which they are uniquely exposed, related to both racial and sexual minoritized status [46-48].

While HIV-related inequities are the most well-studied health concern impacting Black sexual minority men, studies have notably demonstrated that disparities in HIV incidence are not driven by differences in individual-level sexual risk behaviors but are driven in part by differential health care access and concentrated poverty resulting from SRD in the health, housing, and economic sectors [49-52]. While research on SRD is central to understanding the health of Black sexual minority men, such research remains sparse, particularly with respect to health outcomes beyond HIV and sexual health [53].

### Study Setting and Historical Context

Atlanta, Georgia, often referred to as the “de facto capital of the South” [54], provides a unique combination of supportive and detrimental environmental features for Black sexual minority men. In the Southern United States, Atlanta has a substantial population of Black sexual minority men, due in part to the migration of Black sexual minority men seeking economic opportunity and freedom of sexual expression [55,56]. However, Atlanta remains impacted by the legacy of legal racial segregation and Jim Crow policies that followed the abolition of slavery [57,58]. In particular, Black sexual minority men in Atlanta remain vulnerable to multiple forms of SRD, including persistent housing discrimination, increased gentrification, residential segregation based on race or income, and structural homonegativity [10,56,59,60].

### Risk Environment Framework and SRD

**Figure 1:**
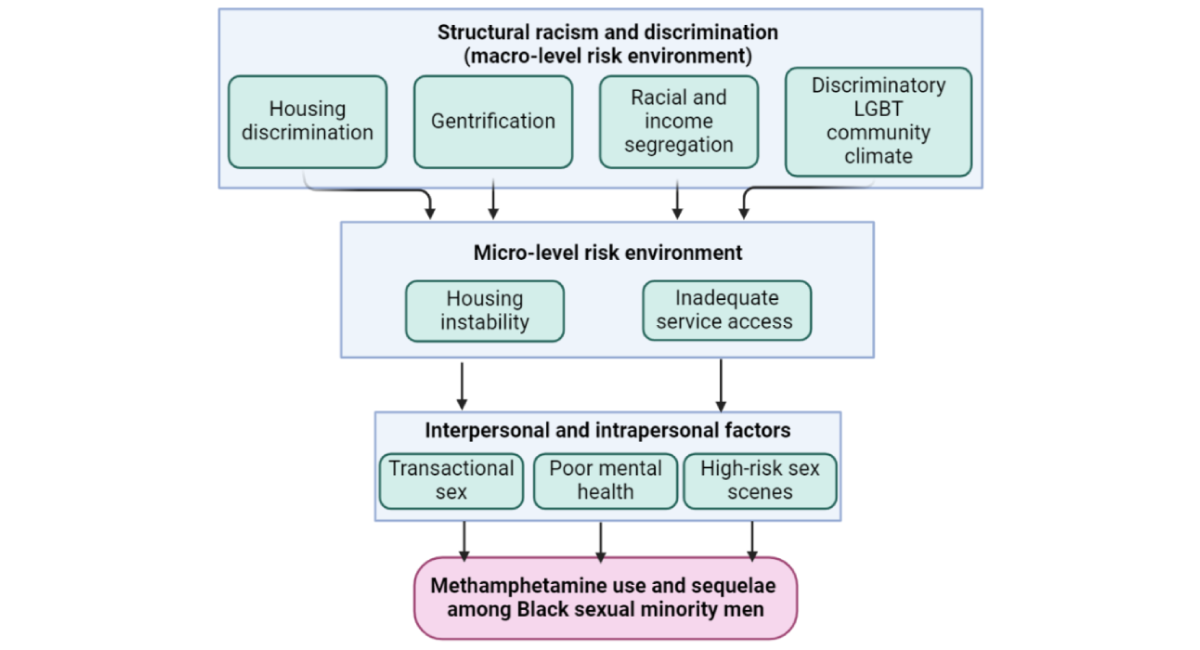
Risk environment framework conceptual model. LGBT: lesbian, gay, bisexual, transgender.

Prior exploratory qualitative work identified housing instability and inadequate access to health and social services as potential pathways through which various forms of SRD may increase the risk for methamphetamine use and its sequelae among Black sexual minority men in Atlanta, Georgia [10]. Grounded in the risk environment framework [61-65], we posit that 4 intersecting forms of SRD in the macro-level risk environment (housing discrimination; gentrification; racial and income residential segregation; and discriminatory lesbian, gay, bisexual, transgender [LGBT] community climate) influence the meso-level problems of housing instability and inadequate service access, ultimately leading to the health outcome of consequent methamphetamine use among Black sexual minority men (Figure 1).

### Study Objectives

The overall goals of this study are to (1) identify structural intervention targets to improve the prevention and treatment of methamphetamine use among Black sexual minority men, and (2) generate knowledge that will inform the design of multilevel, culturally congruent structural approaches for the prevention of methamphetamine use and its associated harms among Black sexual minority men. We seek to address these objectives through three specific aims: (1) to examine the impacts of census tract–level SRD measures on housing instability, service access, and methamphetamine use among Black sexual minority men in Atlanta; (2) to elicit narratives of methamphetamine use, housing instability, service access, and SRD; and (3) to examine systems of structural influence and develop qualitative causal maps linking various forms of SRD, service access, housing, and methamphetamine use. This paper will describe the methods that will be used to achieve these aims.

## Methods

### Study Overview and Design

#### Overview

**Figure 2:**
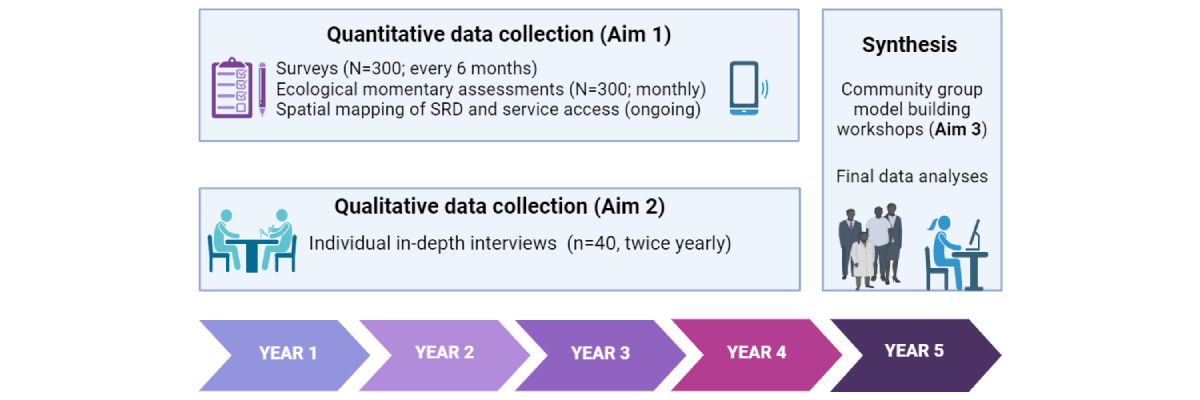
Convergent parallel mixed methods study design. SRD: structural racism and discrimination.

This study uses a convergent, parallel mixed methods design set over a 5-year period (Figure 2), using multiple forms of qualitative and quantitative data via an integrative approach, whereby preliminary quantitative data will inform sampling and interview guide content for in-depth interviews (IDIs), and qualitative data will inform consideration of additional concepts and constructs to surveil via quantitative data collection. Qualitative data analysis will also inform the selection of covariates, potential modifiers, and interpretation of the impact of unmeasured confounders in quantitative analysis. Data sources for the 3 phases of the study (corresponding to our 3 study objectives) are described as follows.

#### Examining the Impacts of Census Tract–Level SRD Measures on Housing Instability, Service Access, and Methamphetamine Use Among Black Sexual Minority Men in Atlanta

We are in the process of recruiting a community-based cohort of Black sexual minority men in Atlanta (N=300), each of whom will be followed for 2 years. Data collection includes (1) self-administered, web-based electronic surveys every 6 months to measure methamphetamine use (alone or in combination with other drugs), residential location, housing instability, mental health indicators, and other covariates; (2) monthly brief assessments of supplementary methamphetamine use and location data via ecological momentary assessment (EMA) methods; (3) abstraction of HIV prevention and treatment-related clinical data from electronic medical records; and (4) census tract–level geolocation of four measures of SRD (housing discrimination, gentrification, racial and income segregation, discriminatory LGBT community climate) and measurement of spatial accessibility of Black sexual minority men to supportive services (substance use treatment and harm reduction services).

### Participant Eligibility

Participants are being screened for eligibility via a self-administered or in-person, web-based survey. Eligibility criteria for enrollment are self-reported (1) Black race, including multiracial identities; (2) male gender identity; (3) identification as gay, bisexual, or any history of consensual sex with men; (4) aged 18-44 years; and (5) current residence within the 5-county Atlanta metropolitan area (Cobb, Clayton, DeKalb, Fulton, and Gwinnett counties).

### Participant Recruitment

#### Overview

Specific strategies for Black sexual minority men recruitment include (1) community-based organization (CBO) recruitment, based on preexisting partnerships with CBOs located in various parts of the Atlanta metropolitan area; (2) web-based recruitment on apps and websites shown to be effective for recruitment of sexual minority men for research [66]; (3) venue-based recruitment, targeting events catering to sexual minority men around Atlanta; (4) clinic-based recruitment at the Grady Ponce De Leon Center, a large HIV clinic serving the entire Atlanta metropolitan area; and (5) research-based recruitment, using lists of patients recruited from previous studies who have expressed interest in being contacted for future studies. We cross-reference lists of potential participants obtained from the various sources mentioned earlier and eliminate duplicates to ensure that individuals are not contacted multiple times.

#### Eliciting Narratives of Methamphetamine Use, Housing Instability, Service Access, and SRD

Via a purposive sampling strategy based on baseline survey data, we are inviting 40 cohort participants to participate in a longitudinal substudy of serial qualitative IDIs conducted every 6-12 months. We will stratify recruitment for this substudy on methamphetamine use to result in 20 participants who use meth, and 20 participants who do not. We will also aim to present diverse perspectives with respect to age range, housing status, and exposure to our 4 SRD measures. We anticipate this sample size will be adequate for thematic saturation [67], breadth and depth of data, and allow for comparative analyses.

#### Group Model–Building Workshop and Development of Causal Maps

In the final year of the study, we will conduct a participatory group model–building workshop with up to 25 participants comprising a mix of four types of community stakeholders: (1) community activists and organizers; (2) CBO staff and leaders; (3) health care and social service providers; and (4) public health and local housing officials. In recruiting participants for this workshop, we will seek diverse representation in terms of age, occupation, experience, and educational background. The workshop will involve a participatory, iterative process that engages stakeholders in creating qualitative causal maps, also known as causal loop diagrams, of the interconnected relationships between SRD, mobility, and methamphetamine use.

### Measures and Procedures

#### Outcomes and Covariates (Including Micro-Level Risk Environment)

Our primary outcome is methamphetamine use. Guided by the World Health Organization’s Alcohol, Smoking and Substance Involvement Screening Test [68], we will combine measures of periods (eg, ever, past year, past 30 days) and frequencies of methamphetamine use to create meth-related risk categories (low-, medium-, or high-risk use). Secondary outcomes will include other drug use, mental health comorbidities, and sexual risk behavior (eg, multiple sexual partners or condomless sex). The micro-level risk environment will be assessed through questions about housing status, perceived neighborhood characteristics, and residential location. Effect modifiers will include additional risk and resilience factors: transactional sex, use of geosocial networking apps, active coping (John Henryism [69]), social support, health care and social service use, and experiences with interpersonal discrimination. Details of these measures are shown in Table 1.

**Table 1:** Individual-level measurement priorities (longitudinal electronic survey domains).

Outcome and construct		Measures
**Primary outcome**		
	Methamphetamine use	NIDA^a^-modified ASSIST^b^ [68], DAST^c^ [70], additional structured questions (frequency and administration routes)
**Secondary outcomes (sequelae of** **methamphetamine** **use)**		
	Other substance use (eg, cocaine, opioids)	NIDA-modified ASSIST, DAST, structured questions (ever, past 6 months, past 30-day, frequency, and administration routes)
	Depression	Center for Epidemiologic Studies Depression Scale [71]
	Anxiety	Generalized Anxiety Disorder-7 Scale [72]
	Stress and trauma	PTSD^d^ Checklist-Civilian [72]
	General well-being	General Well-Being Scale [73]
	HIV risk behavior	Items adapted from HIV Prevention Trials Networks 061 protocol
**Covariates, mediators, moderators (micro-level risk environment, inter- or intrapersonal factors, and resilience factors)**		
	Housing stability (mediator)	Moves in last 6 months, items from Recession and Recovery Study [74]
	Eviction	Series of items used by Desmond et al [75-77]
	Housing conditions	Questions informed by the Department of Housing and Urban Development
	Need for affordable housing	Questions informed by the Picture of Subsidized Households [78]
	Interpersonal discrimination	Everyday discrimination scale [79] Internalized homonegativity inventory [80]
	John Henryism	John Henryism Active Coping Scale [69]
	Social support	Multidimensional scale of personal social support [81]
	HIV prevention or care behaviors	HIV care continuum metrics [82], PrEP^e^ use [83]
	Mental health care use	Selected items from the National Survey of American Life [84]
	Drug treatment use	Selected items from the National Survey of Drug Use and Health
	Mode of transportation	Single item: What is the most common way that you get around the city?
	Perceived neighborhood characteristics	Neighborhood social control scale, neighborhood social cohesion scale, and neighborhood violence scale [85]
	Transactional sex	Items from Bauermeister et al [86] and Stevens et al [87]
	Geosocial network app use	Items from Duncan et al [88]
**Sociodemographics**		
	Age	Single item: What is your date of birth (baseline only)
	Ethnicity	US Census categories (at baseline only)
	Poverty	Material resources scale [89], income, source of income
	Food insecurity	US Department of Agriculture measure of food insecurity [90]
	Education	Single item: What is your highest level of completed education?
	Rent or mortgage payment cost	Single item: What is your current monthly rent or mortgage payment?
	Household size	Single item: How many other people live in your home?

^a^NIDA: National Institute on Drug Abuse.

^b^ASSIST: Alcohol, Smoking, and Substance Involvement Screening Test.

^c^DAST: drug abuse screening test.

^d^PTSD: posttraumatic stress disorder.

^e^PrEP: preexposure prophylaxis.

#### Census Tract–Level Measures of SRD

To measure the macro-level risk environment, we will develop census tract–level proxies for at least 4 constructs of SRD (housing discrimination, gentrification, residential racial and income segregation, and discriminatory LGBT community climate) and potential place-based mediators of the relationships of SRD to methamphetamine use (eg, spatial access to behavioral health and harm reduction services). To develop these measures or proxies, we will extract data from publicly available datasets that include but are not limited to the US Census Bureau’s American Community Survey, US Department of Agriculture Labor Statistics, the Substance Abuse and Mental Health Services Administration, National Survey of Substance Abuse Treatment Services, and AIDSVu. Examples of census tract–level measures of consideration are shown in Table 2, with references to our own or others’ prior research and descriptions of potential means of operationalization of these variables.

**Table 2:** Examples of potential census tract-level measures of structural racism and discrimination and service access.

Construct (exposure, mediator)	Potential data source(s)	Example(s) of prior use
Housing discrimination (exposure)	Housing Mortgage Disclosure Act database (Federal Reserve Board)	Gee [91], Linton et al [92]
Gentrification (exposure)	Geolytics Neighborhood Change Database; American Community Survey	Linton et al [93], Corrigan et al [60]
Residential racial segregation (exposure)	US Census American Community Survey	Linton et al [92], Krieger et al [94]
Residential racial and income segregation (exposure)	US Census American Community Survey	Krieger et al [94]
LGBT^a^ community climate (exposure)	American Community Survey, USDA^b^ Labor Statistics	Oswald et al [95]
Spatial access to substance abuse treatment programs (mediator)	SAMHSA^c^ N-SSATS^d^	Cooper et al [62,96]
Spatial access to harm reduction services (mediator)	AIDSVu	Cooper et al [62,96]

^a^LGBT: lesbian, gay, bisexual, transgender.

^b^USDA: US Department of Agriculture.

^c^SAMHSA: Substance Abuse and Mental Health Services Administration.

^d^N-SSATS: National Survey of Substance Abuse Treatment Services.

#### EMA Surveys

EMA is a smartphone-based data collection tool that can actively capture substance use behaviors and social exposures while also passively collecting location data using GPS tracking [97,98]. Because we expect high participant mobility, we are using monthly EMA check-in surveys to track locations more frequently than semiannual web-based electronic surveys. The brief (approximately 5 minutes) EMA surveys collect data on methamphetamine use, provide opportunities to update contact information, and encourage retention in the study (through frequent, brief, and incentivized engagement). Surveys will be administered monthly over the 2-year duration of the study via a smartphone-based app that will be downloaded to the participants’ phones at the baseline assessment. The EMA app is provided through LifeData, an established Health Insurance Portability and Accountability Act (HIPAA)–compliant software company.

#### Retention Plan

At enrollment, participants will fill out a locator form to document preferences with regard to modes of contact, as well as alternate contacts. To maintain participant engagement for the duration of the study, research staff will send out survey reminders, schedule interviews, troubleshoot participant challenges as appropriate, and mail care packages to participants periodically. We will also periodically check in with participants via an automated text messaging service. Monthly EMA surveys will serve as additional opportunities for engagement; if participants miss an EMA survey, we will contact them to determine individualized strategies to enhance follow-up. Given the criminalization of methamphetamine use, we will scan arrest records and mugshots if needed, as done in our previous research [99-101].

#### Qualitative Interviews

All IDI participants will complete a life-line interview facilitated by a semistructured guide at baseline and then IDIs every 6 months afterward. The life-line interview method is useful for collecting autobiographical data of participants across their life span [102]. Via the life-line interview method, we will obtain information about participants’ insights and experiences with the following domains of our conceptual model (Figure 1): (1) the four forms of SRD; (2) housing; (3) service use and accessibility; (4) interpersonal and intrapersonal factors; and (5) methamphetamine use and sequelae. The baseline life-line interview will also explore participants’ backgrounds, migration histories, and formative experiences. Follow-up IDIs will explore the same domains examined at baseline, using an iterative process guided by responses to the baseline life-line interview, as well as choropleth maps generated from preliminary data.

All interviews will be conducted by experienced study staff trained in qualitative techniques and cultural humility, many of whom are Black and sexual minority men. All IDIs will be digitally recorded, deidentified, and transcribed verbatim.

#### Group Model–Building Workshop

This approach is rooted in community-based system dynamics, a participatory method for involving community stakeholders in understanding and changing systems [103,104]. The design of the workshop will be led by the core modeling team, which will include a subset of members from the research team and community partners. Through a group model–building workshop, we will enable the co-creation of qualitative causal maps that facilitate the visualization of interconnected factors in a complex system and elicit stakeholders’ insights and recommendations to implement systems-level change [104]. We will facilitate the workshop centered around the question, “What barriers and facilitators affect the prevention and treatment of methamphetamine use among Black sexual minority men in Atlanta?” Predetermined, adaptable exercises, or scripts used in previous research [105] will form the basis for workshop activities. Proposed scripts are depicted in Table 3; these will be refined and adapted with our partners and study team prior to implementation. We will use these workshop products to develop community- and research-derived recommendations for community-level action in Atlanta.

**Table 3:** Proposed workshop activities (adapted from Mui et al [105], which is published under Creative Commons Attribution 4.0 International License [106]).

Script	Functions	Activity details	Outputs
1. Setting the stage	Establish the purpose, processes, and ground rules of the workshop	Complete this activity in small groups, then altogether	List of guidelines and priorities
2. Behavior over time graphs	Generate multiple factors as potential drivers of methamphetamine use among Black sexual minority men	Work in pairs, then join a larger group discussion	Candidate factors for causal loop diagrams
3. Dots	Sort through possible choices and select the most important factors related to methamphetamine use among Black sexual minority men	Each participant places up to four sticker dots next to the graphs that they deem to be the highest priority	Prioritized factors
4. Connection circles	Introduce the concepts of causal connections and feedback relationships in a system	Factors identified in scripts (2) and (3) used to develop connection circles, first in small groups and then all together	One connection circle per group
5. Causal loop diagrams	Synthesize multiple perspectives of methamphetamine use and reveal new insights	Connections identified in script (4) expanded upon by including feedback loops and identification of sub-systems	One causal loop diagram per group
6. Action ideas	Identify and prioritize action ideas along a matrix of low-high feasibility and low-high impact	First in small groups, and then in larger group	Prioritized list of potential structural interventions

Throughout the workshop, research assistants will take notes on large flip charts to record concerns and recommendations voiced during the collaborative feedback process. Flipchart pages will become primary data sources. Workshops will also be audio recorded and transcribed for further review. Field notes (with both observational and interpretive elements) will be taken by research assistants observing the workshop sessions. At the end of the workshop, the study team will develop combined reflection notes based on these conversations.

#### Community-Engaged Research Approach

All of the data collection described above will be informed by extensive, ongoing community engagement. We collaborate closely with CBOs that work closely with Black sexual minority men who use or are at risk for using methamphetamine across the Atlanta metropolitan area. Our CBO partnerships enable us to recruit from geographically diverse areas of Atlanta and refer participants to those organizations for support services where indicated. We have also convened an active community advisory board (CAB) of Black sexual minority men, who were recruited primarily through referrals from community partners and selected with the goal of representing Black sexual minority men across a range of social backgrounds. Our CAB meets weekly: activities include reviewing instruments, developing recruitment strategies, and capacity building. Finally, we consult periodically with an expert advisory panel comprised of leading scholar activists in Atlanta’s Black gay community; they provide input at each stage and will facilitate community dissemination of results and translation of our research to practice.

### Data Analysis

#### Quantitative Data Analysis

##### Preliminary Analysis

Across each visit, we will assess the distribution of methamphetamine use, secondary outcomes, and demographic and behavioral characteristics among participants. We will also assess the distribution of participants across census tracts and summarize exposure to census tract–level measures of SRD and spatial access to services. Geolocations of participants across census tracts and census tract–level information will be mapped in ArcGIS (Esri) or R (R Foundation of Statistical Computing) to assess the spatial distribution of participants, census tract–level measures of SRD and other constructs, and access to health care services over time.

Based on the results of pilot spatial analyses of segregation and gentrification in Atlanta and other cities [60,93], we will explore the correlation among housing discrimination, residential segregation and gentrification, LGBT community climate, as well as other census tract–level and spatial access measures in our proposed analyses. If necessary, we will use data reduction techniques including principal component analysis, or we will construct an outcome-predicting census tract score [107], to characterize census tract–level exposures for each year of follow-up.

##### Multilevel and Longitudinal Analyses

We will use random effects models to estimate the longitudinal associations of census tract–level exposure to measures of SRD with methamphetamine use and secondary outcomes. We will adjust for time-dependent and time-independent covariates to describe changes in the population mean given changes in covariates, while accounting for within-census tract nonindependence of observations. Analyses will include random intercepts for census tracts. The distribution and link function of the model will be determined based on the preliminary investigation of the distributions of the measures of methamphetamine use and secondary outcomes. To establish temporality, census tract–level exposure to SRD and covariates will be lagged at least 1 visit, and the suitability of alternative lagging strategies will also be explored. Cross-classification will be used to address any movement of participants across census tracts over time.

##### Mediation Analyses

Adopting the counterfactual approach to mediation [108,109], we will decompose the total association of forms of SRD with methamphetamine use and secondary outcomes into natural direct and indirect effects via primary mediators, housing instability, and health care service access. We will follow the formal mediation analysis procedure by Lange et al [110], which allows for simultaneous examination of multiple mediators, flexible outcome, and mediator specification (binary and categorical; continuous scales and indices will be categorized based on quantiles). The intuition behind the approach is that the analysis is conducted on a weighted pseudopopulation, in which exposure-outcome and mediator-outcome relationships are unconfounded through the application of inverse probability weights. In this study, the hypothesized paths (informed by the literature; to be refined through empirical analysis) that we wish to test are shown in Figure 1.

##### Addressing Missing Data

Data for individual participants may be missing due to loss to follow-up or other reasons, and missingness, if not properly evaluated and addressed, may bias study findings. We will examine whether missingness is associated with observed data using standard methods [111-113]. If we do identify predictors of missingness (ie, data are clearly not missing completely at random), we will conduct a second set of outcome analyses using multiple imputations. As missing data may have different distributions, we will impute using chained equations [114]. We would produce multiple imputed datasets, run outcome analyses on all sets, and derive point and SE estimates for treatment effects and other parameters from the distribution of estimates from the outcome analyses with imputed datasets.

##### EMA Analysis

EMA data will supplement and validate location and substance use data from electronic surveys. This will enable us to take participant mobility between census tracks into consideration when conducting our analyses. Similarly, EMA substance use data that do not match electronic survey responses will prompt us to contact the respondent for clarification or conduct sensitivity analyses without these data. We will also conduct exploratory analyses focused on EMA data, to more fully describe mobility in the cohort and to further explore associations between location and methamphetamine use. Similar to other studies examining longitudinal EMA data [115,116], we will use time-varying effect modeling to examine associations between time-varying, location-related variables (eg, residing in gentrification hotspots) with time-varying methamphetamine use behaviors. Given this flexibility, and that we expect methamphetamine use to switch states (eg, recent use vs none) over time, momentary relationships between location and methamphetamine use can be captured and smoothed over time to produce effect estimates.

#### Qualitative Data Analysis

Qualitative data collection and analysis will be ongoing and iterative. After completion of baseline IDIs, the qualitative analysis team will read all transcripts and establish a codebook comprised of a list of preliminary a priori and emergent codes. Transcripts will be reread to create pattern codes that connect concepts. Analysts will then code all transcripts in pairs, meeting regularly to discuss findings and emerging themes. Analytic activities will be discussed as a team during dedicated meetings, and discrepancies in coding or interpretation will be resolved through consensus. Consistent patterns in meaning, concepts, and themes across interviews will be identified. Comparative analyses will clarify differences that may exist between subgroups of participants (eg, those in highly gentrified vs nongentrified areas). Findings will be reviewed regularly with expert advisory panel and CAB to strengthen representation and transactional validity. Emergent findings from IDIs at a given time may inform changes to guides used in subsequent IDIs.

Our team-based qualitative analysis approach will use an interpretive phenomenological framework [117] to establish experiential commonality among Black sexual minority men with regard to the phenomena of methamphetamine use, gentrification, discriminatory community climate, and housing instability while analyzing emerging patterns, themes, and categories in the data. This approach will allow us to learn about culturally circumscribed behaviors, language, and roles among Black sexual minority men, including individual and shared experiences and meanings related to methamphetamine use, housing, and the various forms of SRD. We will conduct comparative analyses between groups based on methamphetamine use (comparing those who use to those who do not), in order to explore both unique and shared experiences. We will also use aspects of deductive analysis through the creation of a priori codes representing macro- and micro-level constructs within our risk environment model (Figures 1 and 2), as informed by similar previous research (Tables 1 and 2).

#### Group Model–Building Workshop

**Figure 3:**
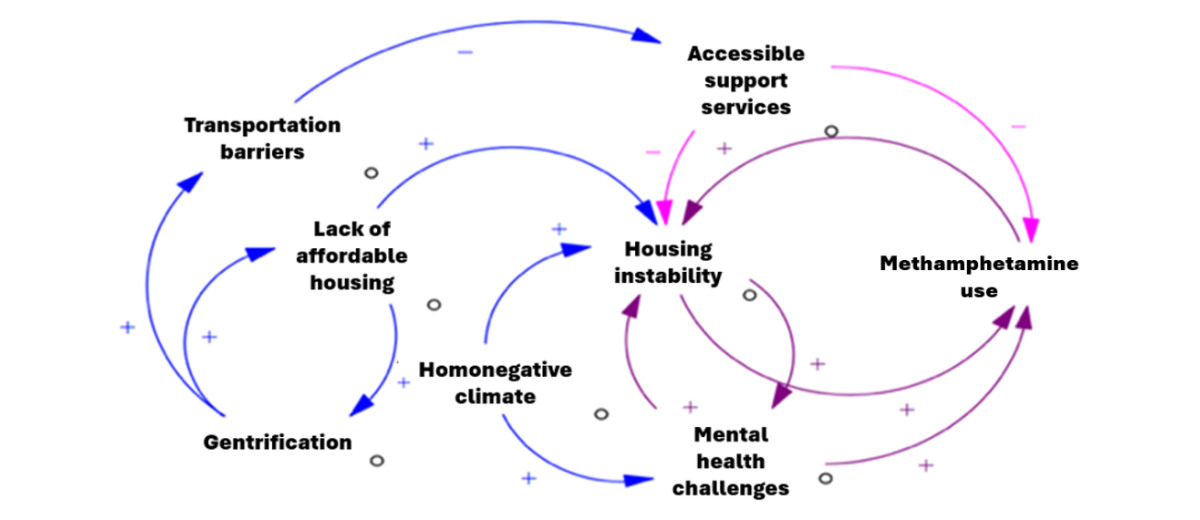
Sample causal loop diagram.

The main product of the workshop will be a qualitative causal loop diagram (example shown in Figure 3) depicting the system of factors influencing the prevention and treatment of methamphetamine use among Black sexual minority men in Atlanta, and an accompanying prioritized list of action recommendations. Through an iterative process, the core modeling team will synthesize outputs from the workshop, using Vensim PLE, supplemented by written notes documenting workshop discussions. The core modeling team will also identify key systems insights, including themes and feedback loops. The causal loop diagram will be presented back to participants in a follow-up workshop as a form of member checking, along with preliminary findings from the surveys and interviews. Additional insights will be incorporated into a final causal loop diagram, which will be used to highlight potential points of structural intervention in the system with low-high feasibility and with high or low impact.

### Ethical Considerations

Approval was obtained from the Emory Institutional Review Board (STUDY00004193) and the Grady Health System Research Oversight Committee, with an agreement for reliance from the Johns Hopkins University institutional review board. As appropriate, modifications or additions to the study will be reviewed as an addendum to the initial protocol. For the purposes of participant recruitment, the study was granted a partial waiver of HIPAA authorization. Written informed consent will be obtained from all participants at the time of enrollment. Participants will be asked for HIPAA authorization for medical record abstraction during the consent discussion at the time of enrollment. Consent forms will be administered in English. Participants will be compensated with US $50 for each completed REDCap (Research Electronic Data Capture; Vanderbilt University) survey and an additional US $10 for each completed monthly brief assessment. Those who participate in the qualitative substudy will receive an additional US $50 per qualitative interview. Participants who opt not to finish the study will be compensated for the surveys or interviews they completed. Data obtained from each participant will be stored confidentially and will only be accessible to authorized research personnel. A unique study identifier will be issued to each enrolled participant and linked to each data entry over the study duration. During future dissemination of study findings, all data will be presented in an aggregate and deidentified format.

## Results

Participant recruitment and data collection commenced in March 2023. As of August 9, 2024, a total of 279 participants had been recruited, with 40 total IDIs completed. Recruitment and data collection are anticipated to be completed by November 2024, with a target of 300 total participants and 40 qualitative substudy participants.

## Discussion

### Expected Findings

The increasing rate of methamphetamine use among Black sexual minority men constitutes a growing manifestation of deep social and health inequities to which this group is uniquely susceptible. The HISTORY project uses an innovative approach aimed at describing this growing problem of methamphetamine use among Black sexual minority men in Atlanta in relation to the spatial distribution of measures of SRD. Upon completion of our aims, we will be able to define potential relationships between SRD and risk for methamphetamine use. Mediation analyses and our qualitative data will allow us to further explore mechanisms linking SRD variables with individual-level methamphetamine use.

### Strengths and Limitations

This multilevel and participatory study, including its qualitative modeling approach, will leverage the experiential knowledge of various stakeholders, proving crucial in the design of specifically tailored interventions for the prevention and treatment of methamphetamine use disorder and its harmful effects among Black sexual minority men. The qualitative approach will further ensure meaningful community engagement by preserving the narratives of participants and key community informants. Despite these strengths, our study also has notable potential limitations. There is a potential for study enrollment to influence key socioeconomic variables, by providing support via the study team and community partner organizations, and also due to financial incentives for participation. There are also likely challenges in retaining the cohort longitudinally. Finally, there may be limited variability in the policy environment given the single-city design. However, given the important questions posed and answered, rigorous and transferable methodology, and wide-reaching results, we anticipate that this study will be used as a basis for further research on locoregional, national, and international scales.

### Future Directions

Beyond what the study will offer for future scientific inquiry, we hope that it will inform the development of programs and policies to improve support for Black sexual minority men who might be at risk for meth-related harms. Future studies could also include quantitative simulation studies based on causal relationships found in our study and exploration of similar themes across different US cities and regions.

### Dissemination Plan

The group model–building workshops will inherently serve as opportunities for dissemination of our qualitative and quantitative findings, as they will bring together community stakeholders to share findings and discuss implications and next steps. Further dissemination of our study results will include presentations at both scientific and program-focused conferences, publication of papers in academic journals, and community-focused presentations to share interim and final results.

## Appendix

Multimedia Appendix 1 Peer-review report by the National Institute on Minority Health and Health Disparities Special Emphasis Panel - Impact of Structural Racism and Discrimination on Minority Health and Health Disparities - National Institute on Minority Health and Health Disparities (National Institutes of Health, USA).

## Abbreviations

CAB: community advisory board
CBO: community-based organization
EMA: ecological momentary assessment
HIPAA: Health Insurance Portability and Accountability Act
HISTORY: Health Impacts and Struggles to Overcome the Racial Discrimination of Yesterday
IDI: in-depth interview
LGBT: lesbian, gay, bisexual, transgender
REDCap: Research Electronic Data Capture
SRD: structural racism and discrimination

## Data Availability

The datasets generated and analyzed during this study will be available upon request from the corresponding author after the completion of our study aims.
