# Supplementary material for: Structural Influences on Methamphetamine Use Among Black Sexual Minority Men (HISTORY Study): Protocol for a Longitudinal Cohort Study
Source: JMIR Res Protoc. 2024 Oct 31;13:e63761. doi: 10.2196/63761 (PMC11565090; doi:10.2196/63761)
Supplement: Multimedia Appendix 1 [file resprot_v13i1e63761_app1.pdf]

SUMMARY STATEMENT

PROGRAM CONTACT: (Privileged Communication) *Release Date:* 12/10/2021
[redacted]
Revised Date:

Application Number: 1 R01 DA056235-01

Principal Investigators (Listed Alphabetically):

HUSSEN, SOPHIA A. (Contact)
LINTON, SABRIYA L

Applicant Organization: EMORY UNIVERSITY

Review Group: ZMD1 KNL (J1)
National Institute on Minority Health and Health Disparities Special Emphasis Panel
Impact of Structural Racism and Discrimination on Minority Health and Health Disparities

Meeting Date: 11/15/2021
Council: JAN 2022
Requested Start: 04/01/2022
RFA/PA: MD21-004
PCC: EB/SKD
Dual IC(s): AA, AG, AI, DC, DE, DK, EB, ES, EY, GM, HD, HL, MD, MH, NR

Project Title: Structural Influences on Methamphetamine Use among Black Gay and Bisexual Men in Atlanta
SRG Action: Impact Score:22
Next Steps: Visit https://grants.nih.gov/grants/next_steps.htm
Human Subjects: 30-Human subjects involved - Certified, no SRG concerns
Animal Subjects: 10-No live vertebrate animals involved for competing appl.
Gender: 3A-Only men, scientifically acceptable
Minority: 2A-Only minorities, scientifically acceptable
Age: 7A-Only Adults, scientifically acceptable

Project Year	Direct Costs Requested	Estimated Total Cost
1	[redacted]	[redacted]
2		
3		
4		
5		
TOTAL	[redacted]	[redacted]

EARLY STAGE INVESTIGATOR
NEW INVESTIGATOR

1R01DA056235-01 Hussen, Sophia

EARLY-STAGE INVESTIGATOR
NEW INVESTIGATOR

RESUME AND SUMMARY OF DISCUSSION: This application is submitted in response to RFA-MD-21-004 “Understanding and Addressing the Impact of Structural Racism and Discrimination on Minority Health and Health Disparities (R01). In this application the Investigators propose to examine the impacts of census-tract level measures of structural racism and discrimination (SRD) on housing instability, service access and meth use among Black gay, bisexual and other men who have sex with men (GBMSM) in Atlanta, to elicit narratives of meth use, housing instability, service access and SRD among Black GBMSM and to examine systems of structural influence and develop qualitative causal maps linking various forms of SRD, service access, housing and meth use among Black GBMSM. The review panel members agreed that the proposed study is significant, and if successful, findings may have the potential to increase the knowledge about individual and structural risk factors that influence meth use among GBMSM and to inform future prevention interventions. The investigative team is outstanding with complementary and multidisciplinary expertise. A few relatively minor addressable concerns regarding the approach were discussed including lack of detail about the framework that guides the qualitative analysis and lack of clarity about how the ecological momentary assessment data will be used. In addition, little detail is provided about differences between people with meth disorders and people who are experimenting with meth. Overall, the proposed research is potentially very important and likely to have high overall impact on the field.

DESCRIPTION (provided by applicant): Emerging data demonstrates a rise in methamphetamine (meth) use among Black gay, bisexual and other men who have sex with men (GBMSM). Meth is associated with myriad physical and social harms, and has the potential to exacerbate existing health inequities impacting Black GBMSM. Existing meth prevention and treatment interventions do not address the unique social position of Black GBMSM or the related health impacts of structural racism and discrimination (SRD). Based on our preliminary studies, we hypothesize that four forms of SRD are likely to influence meth use among Black GBMSM: housing discrimination, gentrification, racial and income segregation, and discriminatory LGBT community climate. Additionally, we posit that these forms of SRD operate through housing instability and inadequate service access to increase risk for meth use among Black GBMSM. The goal of this project is to generate knowledge that will inform the design of multi- level, culturally congruent structural approaches to prevent meth use and associate harms among Black GBMSM. Our study will be based in Atlanta, Georgia – an ideal setting to examine these questions given the cultural significance for Black Americans, large population of Black GBMSM, and recent SRD-related demographic shifts. Drawing on the risk environment framework as its theoretical foundation, this study will pursue three specific aims: (1) to examine the impacts of census-tract level measures of SRD on housing instability, service access and meth use among Black GBMSM in Atlanta; (2) to elicit narratives of meth use, housing instability, service access and SRD among Black GBMSM; and (3) to examine systems of structural influence and develop qualitative causal maps linking various forms of SRD, service access, housing and meth use among Black GBMSM. For the first aim, we will recruit N=300 Black GBMSM into a longitudinal cohort study, and conduct serial surveys every 6 months over a two-year follow-up period. Additional location and meth use data will be obtained monthly using ecological momentary assessment technology. Individual survey responses will be linked to geolocated census-tract level measures of SRD and service access. For the second aim, we will conduct a longitudinal qualitative study including in-depth interviews and walking ethnographies with N=40 cohort participants, to gather insights on their experiences with SRD, housing, service access and meth use. For the third aim, we will use a community-based system dynamics approach to convene a group model building workshop with key stakeholders (N=25), which will lead directly to the

development of causal maps that will inform structural interventions. The proposed research is highly significant because of its potential to inform effective structural strategies for preventing meth use and ameliorating meth-related harms among Black GBMSM.

PUBLIC HEALTH RELEVANCE:
Methamphetamine use is an emerging threat to the health and well-being of Black gay, bisexual and other men who have sex with men (GBMSM). The proposed project will examine pathways between four forms of structural racism and discrimination (housing discrimination, gentrification, racial and income segregation, and discriminatory community climate for lesbian, gay, bisexual and transgender individuals) and methamphetamine use in this population. This information will be used to inform structural interventions to prevent methamphetamine use and related harms among Black GBMSM.

CRITIQUE 1

Significance: 2
Investigator(s): 2
Innovation: 2
Approach: 1
Environment: 2

Overall Impact: This application documented the rise in meth use among GBMSM in Atlanta and proposed to examine this rise using an innovative strategy that considers how housing, discrimination, gentrification, income segregation, and discriminatory LGBT community climate impacts SU. The proposal will examine census tract level measures, elicit narratives for meth use, and longitudinally enroll 300 GBMSM to examine systems of structural influence. At the end of the research activities, the applicant will, in partnership with key constituent groups, develop causal maps that will inform structural interventions with GBMSMs.

1. Significance:

Strengths

- The investigators underscored the negative health experiences of GBMSMs.
- They also highlighted the increasing rates of meth use and negative health sequala.
- The investigators refocused our attention to the structural factors that, in partnership with the individual factors, influence this increase in use.
- This research is theoretically grounded in the risk environment framework.

Weaknesses

- Considering how some of the historical experiences of GBMSM may independently impact the structural factors being examined may have brought to divergent lines of research together and move the field more forward.

2. Investigator(s):

Strengths

- The investigators appear to have a good collaborative relationship
- The investigators academically appear well suited to carry out this research.

- The proposed investigators all present with complementary skills to this research.

Weaknesses

- There are a lot of investigators included.

3. Innovation:

Strengths

- The use of walking ethnographic is innovative.
- The integration of system dynamics is also innovative.

Weaknesses

- None noted.

4. Approach:

Strengths

- The approach is articulated well
- The proposed research considers the counties in Atlanta and unique challenges and rewards that each brings.
- The approach considers how best to engage and track study participants.

Weaknesses

- The inclusion of the expert panel could have been more centrally included in the design.

5. Environment:

Strengths

- The environment appears to be well suited to support the proposed research
- The academic and community partners referenced all appear supportive to the research proposed.

Weaknesses

- None noted.

Protections for Human Subjects

Acceptable Risks and/or Adequate Protections

- The protections proposed appear appropriate.

Data and Safety Monitoring Plan (Applicable for Clinical Trials Only):

Acceptable

- o Safety protocols appear appropriate.

Inclusion Plans

- Sex/Gender: Distribution justified scientifically
- Race/Ethnicity: Distribution justified scientifically

- Inclusion/Exclusion Based on Age: Distribution justified scientifically

Vertebrate Animals
Not Applicable (No Vertebrate Animals)

Biohazards
Not Applicable (No Biohazards)

Select Agents
Not Applicable (No Select Agents)

Resource Sharing Plans
Acceptable

Authentication of Key Biological and/or Chemical Resources
Not Applicable (No Relevant Resources)

Budget and Period of Support
Recommend as Requested

CRITIQUE 2

Significance: 2
Investigator(s): 2
Innovation: 2
Approach: 3
Environment: 1

Overall Impact: This project proposes a mixed-methods longitudinal test of the hypothesis that structural racism and discrimination (SRD) is predictive of Methamphetamine use by Black GBMSM with housing instability and inadequate access to health/social services as mediators, using survey (repeated every 6 months for 2 years), ecological momentary assessment (EMA), and archival census tract- level data for quantitative analyses and narrative qualitative interview data. Results will be used in an innovative participatory group model building workshop with activists, community-based organizations, and service providers to develop causal maps and policy-relevant action priorities for structural interventions. The combination of focused hypotheses and quantitative measures with extensive community participatory input provide a strong basis for actionable findings for a public health problem that affects a very high-risk sub-population subject to intersectional SRD and homophobic bias. The study team has the necessary scientific expertise and community access and connections to complete the proposed project successfully. Limitations are potentially resolvable, including insufficient detail on complex statistical analyses, how more complex pathways from SRD to meth use will be handled, and generalizability of results to other sites.

1. Significance:

Strengths

- Methamphetamine use by Black GBMSM is an increasingly severe public health problem, but research on its determinants is sparse and focused on individual-level risk factors, and neither addresses the intersection of structural racism and discrimination (SRD) nor has led to prevention interventions other than generic individual-level behavior change models.
- Black GBMSM are a very high-risk sub-population subject to the synergistic adversity of intersectional SRD and homophobic bias for whom structural/policy interventions are needed in order to prevent significant medical and behavioral health morbidity and death.
- Housing and services access disparities due to racism, gentrification, segregation, and discrimination against LGBTQ persons are potentially modifiable structural factors that do not place a burden on individuals, the community, or the health/social services system.

Weaknesses

- Whether the findings can be generalized to other GBMSM populations and to areas other than Atlanta without repeating the extensive and lengthy study procedures is uncertain.

2. Investigator(s):

Strengths

- Overall, this is a very strong investigator team with a track record of productivity and collaboration on complementary NIH-funded studies in the project's area of focus.
- MPI Hussen MD is PI/MPI on 4 NIH-funded public health HIV-focused survey, qualitative interview, and ecological momentary assessment (EMA) research studies with Black gay/bisexual (GBMSM) men with a strong community participatory focus/network.
- MPI Linton PhD is a productive early career social epidemiologist and PI on R03 and R21 studies on HIV, substance use, and neighborhood micro and macro-level factors in the current project site (Atlanta) and her current academic site (Baltimore).
- Co-I Crawford is a social epidemiologist with expertise on HIV risks and racial inequities.
- Co-I Leong is a spatial modeling statistician and ongoing collaborator with MPI Husen.
- Co-I Holland is an infectious disease physician specializing in HIV treatment in Attlanta.
- Co-I Mui is a system dynamic modeling and urban policy development expert.
- Consultants bring expertise and access to key community sites and populations.

Weaknesses

- Consultation from a statistician with expertise in the modeling of complex structural pathways with repeated measures and multiple indicators may be needed

3. Innovation:

Strengths

- The participatory group model building workshop with activists, community-based organizations, and service providers to develop causal maps and policy-relevant action priorities for structural interventions is very innovative.

- The parallel mixed methods qualitative/quantitative design is moderately innovative because of the strong participatory emphasis and narratives by persons with lived experience throughout the project rather than only in the initial exploratory phase.
- Use of EMA data to validate self-report data is moderately innovative.

Weaknesses

- The hypothesized mediation model does not address potential alternative pathways and SRD factors (e.g., disparities related to employment or income) and mediators (e.g., access to recreational, faith, and cultural activities that are not associated with meth use), nor their potential interactions (e.g., housing discrimination x prejudice against GBMSM).
- The proposed mental health measures are potentially relevant but are not justified based on a relationship to SRD or to the outcome of meth use.

4. Approach:

Strengths

- Exploratory qualitative studies at this site have identified housing instability, inadequate access to health/social services as potential mediators of a SRD-Meth Use relationship.
- Pilot research by MPI Linton used quantitative special analysis methods to identify Black-White racial differences in structural predictors of STI use by GBMSM in the study sites.
- Participatory qualitative methods are proposed to ground the study in lived experiences, guided by an expert advisory panel of locally networked scholar activists and a standing community advisory board with 8 Black GBMSM.
- Partnerships with two community-based organizations (H2L, PIHC) facilitate recruitment and retention of the large (N=300) survey cohort of Black GBMSM.
- Well selected and justified validated self-report measures are proposed for outcomes, mediators, moderators, and covariates – with validation of the key location and substance use data by monthly collection of EMA data.
- Careful thought has been given to potential challenges to enrollment, retention, and possible alternative pathways to meth use that may be identified in qualitative interviews.

Weaknesses

- The procedures and guiding framework for qualitative analyses are not described in detail.
- How the multiple data variables will be transformed into the latent variables shown in the mediation model Figure 1) is not described.
- The conceptual model seems better tested by a structural model approach to statistical analyses than the proposed generalized estimating equations modeling proposed.
- The power calculations are based on bivariate tests that do not explicitly take into account the multiplicity of variables and paths being tested.
- Exactly how the EMA data will be used to validate survey data is not described.
- Why the DAST is being used when the ASSIST covers the same ground is not clear.

5. Environment:

Strengths

- The primary host organizations (Emory, Johns Hopkins) have excellent scholarly, IT, and logistical resources for the project and strong connections to relevant community groups.

Weaknesses

- None identified.

Protections for Human Subjects

Acceptable Risks and/or Adequate Protections

Data and Safety Monitoring Plan (Applicable for Clinical Trials Only):

Acceptable

- o IRB and DSMP plans acceptable

Inclusion Plans

- Sex/Gender: Distribution justified scientifically
- Race/Ethnicity: Distribution justified scientifically
- For NIH-Defined Phase III trials, Plans for valid design and analysis: Not applicable
- Inclusion/Exclusion Based on Age: Distribution justified scientifically
- Inclusion of relevant sub-groups is acceptable

Vertebrate Animals

Not Applicable (No Vertebrate Animals)

Biohazards

Not Applicable (No Biohazards)

Select Agents

Not Applicable (No Select Agents)

Resource Sharing Plans

Acceptable

- Plans for sharing resources amongst the investigators' sites are acceptable.

Authentication of Key Biological and/or Chemical Resources

Not Applicable (No Relevant Resources)

Budget and Period of Support

Recommend as Requested

CRITIQUE 3

Significance: 1
Investigator(s): 1
Innovation: 3
Approach: 5
Environment: 1

Overall Impact: The application, Structural Influences on Methamphetamine Use among Black Gay and Bisexual Men in Atlanta, is intended to generate knowledge that will inform the design of multilevel, culturally congruent structural approaches to prevent meth use and associate harms among Black GBMSM. This observational study is likely to provide a foundational level of understanding the role of structural racism and discrimination (SRD) in causing and sustaining methamphetamine use and abuse among Black GBMSM. In the opinion of this reviewer, this study, as it is designed, it is likely to have a moderate public health impact or in addressing SRD in a comprehensive manner. The study provides a justification for why the SRD and methamphetamine use needs to be studied. The investigative team is excellent and has the expertise and capacity to conduct the study has designed. The study also involves key collaborations with relevant organizations. Yet, in spite of numerous strengths, the study design weaknesses decrease the enthusiasm in the study having a meaningful impact in the intersecting epidemics of meth use/abuse and SDR among Black GBMSM.

1. Significance:

Strengths

- Rise in methamphetamine (meth) use among Black gay, bisexual and other men who have sex with men (GBMSM).
- Syndemic effect of meth to exacerbate existing health inequities impacting Black GBMSM.
- There are major gaps: Existing meth prevention and treatment interventions do not address the unique social position of Black GBMSM or the related health impacts of structural racism and discrimination (SRD).
- SRD hypothesized influences on meth use among Black GBMSM: housing discrimination, gentrification, racial and income segregation, and discriminatory LGBT community climate.
- SRD operate through housing instability and inadequate service access to increase risk for meth use among Black GBMSM.

Weaknesses

- None noted.

2. Investigator(s):

Strengths

- Outstanding team of investigators: Hussen (MPI) (expertise in interventions around mental health, HIV and resilience among Black GBMSM in Atlanta; Linton (MPI) (expertise in socio-contextual determinants of substance use and HIV, including extensive experience leading research investigating the interrelationships of community development, gentrification, housing, substance use and HIV risk); Crawford (co-I) (expertise: social epidemiologist, ecological momentary assessment [EMA]) Holland (co-I) Chief Clinical Officer for medical and preventive services at the Fulton County Health Department; Leong (co-I) biostatistician with expertise in

applying geospatial and multilevel modeling, Mui (co-I) (expertise: intersection of community health and urban planning).

- Expert Advisory Panel (EAP): Dr. Malebranche; Smith (Director of the Campaign to End AIDS for the Atlanta metropolitan area); Stephens (Executive Director of the Counter-Narrative Project (CNP).
- CAB with Community Partner Organizations: Here's to Life, Inc.; Positive Impact Health Centers (PIHC)
- Both MPI have the necessary expertise and complimentary elements to carry out the study has designed: Hussen MPI has led multiple R34 studies and one U01; and a total of 42 publications in the NCBI; Linton MPI has led an R21 and multiple prestigious private foundation studies; with a total of 36 publications.

Weaknesses

- None noted.

3. Innovation:

Strengths

- Longitudinal, mixed-methods studies to systematically investigate the impacts of SRD on meth use among Black GBMSM.
- Study will advance knowledge on the public health impact of two underutilized measures of structural racism (i.e., gentrification) and structural homonegativity (i.e., LGBT community climate).
- Unique approach to elicit systems and structural insights that can directly inform knowledge translation, by implementing community-based system dynamics.

Weaknesses

- No major weaknesses noted.

4. Approach:

Strengths

- Overall feasible, adequate and rigorous methodology for Protocol for Aim 1: to examine the impacts of census-tract level measures of SRD on housing instability, service access and meth use among Black GBMSM in Atlanta.
- Strong recruitment strategy for Aim 1 (maps of segregation and gentrification generated by MPI Linton's prior research, and conduct windshield tours, i.e., systematic audits of community resources by visual assessment from view of a vehicle).
- Solid measures of macro SDR: Census Tract-Level Measures of Structural Racism and Discrimination and Service Access.
- Adequate and responsive retaining strategy: Ecological momentary assessment (EMA).
- Overall feasible, adequate and rigorous methodology for Protocol for Aim 2: to elicit narratives of meth use, housing instability, service access and SRD among Black GBMSM.
- Major strength is the use prospective walking ethnographies, comparing the impacts of SDR between users and non-users.
- Strong methodology for gathering, storing and managing the analyses of qualitative data.

- Protocol for Aim 3: to examine systems of structural influence and develop qualitative causal maps linking various forms of SRD, service access, housing and meth use among Black GBMSM; particularly the use of *Community-based system dynamics in the integration of data.*

Weaknesses

- Minor Protocol Aim 1: It would have been good to expand on the strategies for decomposing the total association of forms of SRD with meth use and secondary outcomes into natural direct and indirect effects via primary mediators, housing instability and healthcare service access; and the specific hypotheses that will be tested. A particular concern is whether there will be enough variance in SDR census track level data.
- Minor Protocol Aim 1: Further information is needed on the outcome variable examination period. In the power calculation for sample size, 30% is reported as the prevalence of meth use, but is that an annual prevalence, lifetime or the past 30 days.
- Minor Protocol Aim 2: What is the level of usage of meth to classify as having used or not meth in the eligibility criteria for this protocol.
- The qualitative themes explored in Aim 2 seem superficial (e.g., personal perspectives on SDR) and a missed opportunity to conduct an in-depth qualitative examination on participants lived experiences (not perspectives), their negotiations and, most importantly the qualitative, contextual, situational linkages to meth use (or severity of meth use). Presumably non-users in the sample might have interpersonal and or community assets that users do not have. Conversely what factors distinguish user vs. nonuser if both individuals GBMSM under the same SDR circumstances.

5. Environment:

Strengths

- Outstanding research environment that will support the proposed study as designed.

Weaknesses

- None noted.

Protections for Human Subjects

Acceptable Risks and/or Adequate Protections

- No comments.

Data and Safety Monitoring Plan (Applicable for Clinical Trials Only):

Not Applicable (No Clinical Trials)

Inclusion Plans

- Sex/Gender: Distribution justified scientifically
- Race/Ethnicity: Distribution justified scientifically
- For NIH-Defined Phase III trials, Plans for valid design and analysis: Not applicable
- Inclusion/Exclusion Based on Age: Distribution justified scientifically
- No comments.

Vertebrate Animals
Not Applicable (No Vertebrate Animals)

Biohazards
Not Applicable (No Biohazards)

Select Agents
Not Applicable (No Select Agents)

Resource Sharing Plans
Not Applicable (No Relevant Resources)

Authentication of Key Biological and/or Chemical Resources
Not Applicable (No Relevant Resources)

Budget and Period of Support
Recommend as Requested

THE FOLLOWING SECTIONS WERE PREPARED BY THE SCIENTIFIC REVIEW OFFICER TO SUMMARIZE THE OUTCOME OF DISCUSSIONS OF THE REVIEW COMMITTEE, OR REVIEWERS' WRITTEN CRITIQUES, ON THE FOLLOWING ISSUES:

PROTECTION OF HUMAN SUBJECTS: ACCEPTABLE

INCLUSION OF WOMEN PLAN: ACCEPTABLE

INCLUSION OF MINORITIES PLAN: ACCEPTABLE

INCLUSION ACROSS THE LIFESPAN PLAN: ACCEPTABLE

COMMITTEE BUDGET RECOMMENDATIONS: The budget was recommended as requested.

Footnotes for 1 R01 DA056235-01; PI Name: Hussen, Sophia A.

NIH has modified its policy regarding the receipt of resubmissions (amended applications).See Guide Notice NOT-OD-18-197 at https://grants.nih.gov/grants/guide/notice-files/NOT-OD-18-197.html. The impact/priority score is calculated after discussion of an application by averaging the overall scores (1-9) given by all voting reviewers on the committee and multiplying by 10. The criterion scores are submitted prior to the meeting by the individual reviewers assigned to an application, and are not discussed specifically at the review meeting or calculated into the overall impact score. Some applications also receive a percentile

ranking. For details on the review process, see http://grants.nih.gov/grants/peer_review_process.htm#scoring.
